# The role of adherens junction proteins in the regulation of insulin secretion

**DOI:** 10.1042/BSR20170989

**Published:** 2018-03-21

**Authors:** Waruni C. Dissanayake, Brie Sorrenson, Peter R. Shepherd

**Affiliations:** 1 Department of Molecular Medicine and Pathology, The University of Auckland, Auckland, New Zealand; 2 Maurice Wilkins Centre for Molecular Biodiscovery, The University of Auckland, Auckland, New Zealand

**Keywords:** alpha-catenin, beta-catenin, E-cadherin, insulin secretion, N-cadherin

## Abstract

In healthy individuals, any rise in blood glucose levels is rapidly countered by the release of insulin from the β-cells of the pancreas which in turn promotes the uptake and storage of the glucose in peripheral tissues. The β-cells possess exquisite mechanisms regulating the secretion of insulin to ensure that the correct amount of insulin is released. These mechanisms involve tight control of the movement of insulin containing secretory vesicles within the β-cells, initially preventing most vesicles being able to move to the plasma membrane. Elevated glucose levels trigger an influx of Ca^2+^ that allows fusion of the small number of insulin containing vesicles that are pre-docked at the plasma membrane but glucose also stimulates processes that allow other insulin containing vesicles located further in the cell to move to and fuse with the plasma membrane. The mechanisms controlling these processes are complex and not fully understood but it is clear that the interaction of the β-cells with other β-cells in the islets is very important for their ability to develop the appropriate machinery for proper regulation of insulin secretion. Emerging evidence indicates one factor that is key for this is the formation of homotypic cadherin mediated adherens junctions between β-cells. Here, we review the evidence for this and discuss the mechanisms by which these adherens junctions might regulate insulin vesicle trafficking as well as the implications this has for understanding the dysregulation of insulin secretion seen in pathogenic states.

## Introduction to insulin secretion

Diabetes is essentially a disease caused by loss of the ability to control glucose metabolism and this is ultimately due to a failure to release enough insulin [1]. This is the result of defects in β-cell function as this highly specialized cell type is the sole cell type in the body capable of the production and release of appropriate amounts of insulin to acutely control glucose homeostasis [2]. Type-1 diabetes is characterized by outright loss of β-cells, often due to autoimmune destruction of these cells [1], while type-2 diabetes is characterized by slow loss of β-cell function over time through mechanisms that are less well understood [3,4]. Therefore, there has been intense interest in understanding the molecular mechanisms controlling the release of insulin from β-cells and how these are altered in disease states.

Significant progress has been made in understanding the processes regulating insulin secretion. The initial stages of insulin secretory granule production have been reviewed elsewhere [3,4] and here we focus on the later steps regulating access of these vesicles to the plasma membrane and the subsequent secretion of insulin. Microtubules and the actin cytoskeleton play important roles in these processes. They play positive roles by providing tracks to facilitate the movement of insulin secretory vesicles towards the plasma membrane. However, they ultimately also play roles in limiting access of the granules to the plasma membrane and/or controlling the final movement of vesicles to the plasma membrane, as nicely demonstrated in a total internal reflectance fluorescent (TIRF) microscopy analysis of the dynamics of movement of insulin containing vesicles near the plasma membrane [5,6]. It has been proposed that microtubules create dense structures on which the vesicles move without actually reaching the membrane until appropriate stimuli cause changes in the microtubule structures to allow the vesicles to escape these futile loops [7]. Many studies have also shown that actin negatively regulates the access of insulin granules to the cell periphery under resting state conditions [8,9]. Ultrastructural analysis indicates that β-cells have a dense web of cortical beneath the cell membrane, which may block the access of insulin granules to the cell periphery [10,11]. Evidence indicates that glucose and other insulin secretagogues are involved in remodelling actin near the plasma membrane to allow some insulin granules to access the plasma membrane [8,9]. Once at the plasma membrane, the requisite trigger for insulin release is a glucose mediated influx of Ca^2+^ (reviewed in [3,4]). The influx of Ca^2+^ activates synaptotagmins that in turn activate the fusion of insulin containing vesicles with the plasma membrane through a classical v-SNARE/t-SNARE mediated mechanism [3]. The actin cytoskeleton also appears to control access to the plasma membrane of some components of these SNARE complexes [9].

The process of glucose stimulated insulin secretion (GSIS) is biphasic in nature [12]. The two phases of insulin secretion can be explained by the ‘storage-limited model’, which suggests the presence of different pools of insulin granules within the cell. The first phase of insulin secretion is thought to be due to a ‘readily releasable pool’ of insulin granules fusing with the plasma membrane [2]. This process occurs within 3–10 min of glucose stimulation causing rapid, transient insulin granule exocytosis [12] and approximately 50–200 granules from a total of approximately 10,000 insulin granules in β-cells are released during this first phase of insulin secretion [13,14]. The first phase of insulin secretion only requires an influx of Ca^2+^ triggered physiologically by glucose or artificially by membrane depolarization with agents such as KCl or sulphonylurea drugs [4]. During the second phase of insulin release, insulin granules that are located deep inside the cells, in what is known as the ‘storage granule pool’, translocate to the cell periphery. It is still a subject of debate as to whether these directly replenish the ‘readily releasable pool’ [15] or fuse with the plasma membrane by alternative mechanisms [2]. The second phase insulin secretion can last for several hours if glucose stimulation persists and is considered a slower, more sustained process. During the second phase of insulin secretion, 5–40 insulin granules/minute are released and over time this has a major contribution to the overall amount of insulin secreted [13]. Impairment of the first phase of insulin secretion has long been recognized as an early indication of β-cell dysfunction during type-2 diabetes [16–18]. However, recently it has been shown that the second phase of insulin secretion is also impaired during type-2 diabetes [19].

## The impact of cell polarization on β-cell function

*In vivo* there is now strong evidence that the membranes of β-cells are divided into several distinct domains and maintaining this configuration is critical for proper regulation of insulin secretion by glucose [20,21]. Many β-cells have two connections to blood vessels and it has been speculated that one may represent an arteriole and the other a vein [20,21]. It has been reported that the majority of insulin secretion is in the vicinity of these basal domains in β-cells [20], thus facilitating the rapid release of insulin into the blood stream. By localizing near blood vessels, these membranes are also positioned next to basal lamina allowing the β-cells to form focal adhesion complexes. As reviewed in detail elsewhere, there is strong evidence that the regulation of proteins in these focal adhesions plays a crucial role in regulating cortical actin dynamics and in regulating GSIS [8,22].

The lateral domains of the β-cells face other β-cells and have separate functions to the basal domain with, for example, the bulk of the GLUT2 glucose transporters and calcium channels being in these lateral domains [20,21]. Cadherin mediated adherens junctions are also found in these lateral surfaces of the cells [20,21]. Cadherins are transmembrane glycoproteins that mediate calcium dependent cell–cell adhesion through interaction of their extracellular domain with homotypic cadherins on adjacent cells [23]. E-cadherin and N-cadherin are widely expressed cadherins and are particularly responsible for the formation of the adhesions mediated by adherens junctions [24,25]. These play an important role in regulating the junctions in endothelial and epithelial cells at the zona-adherens and these cells are located in belts that ring the cells in these junctional areas [24]. Adherens junctions are often thought of as structural components of cells required to lock them in their proper place but evidence is now emerging that these junctions play an important role in regulating insulin secretion from β-cells. Adherens junctions were first implicated in regulating insulin secretion over 25 years ago [26]. More recently, this has been supported by several studies. For example, an antibody to the extracellular domain of E-cadherin attenuates GSIS in islets [27]. Further, the loss of E-cadherin in mice seriously impacts on the ability of glucose to stimulate secretion of insulin from islets and results in impaired glucose tolerance in the face of normal insulin sensitivity [28]. These effects are unlikely to be due to major disruptions of cell morphology as the structure of cells in the islet appeared normal and are also unlikely to be due to reduced β-cell number as the loss of E-cadherin actually increased β-cell proliferation in these mice [28]. It was suggested that the effects on proliferation were due to a release of β-catenin from the E-cadherin allowing it to move to the nucleus and increase expression of cell cycle genes. This suggests that the effects of E-cadherin loss on GSIS are more likely due to alterations in either the glucose sensing mechanisms in β-cells or the mechanisms regulating insulin secretory granule trafficking or fusion with membranes. It seems that not only E-cadherin but also N-cadherin is involved in these processes as islets lacking N-cadherin have a greatly reduced number of insulin secretory granules [29]. More direct evidence for a role of cadherins in GSIS in β-cells comes from recent studies that show that there is an increase in the degree to which glucose stimulates insulin secretion when isolated pancreatic β-cells are cultured on plates coated with the extracellular domains for either N-cadherin or E-cadherin to artificially induce cells to form adherens junction structures [30]. Furthermore, the cell surface expression level of E-cadherin is correlated with insulin secretion capability and glucose stimulation increases the surface level of E-cadherin in β-cells [31]. Together, this suggests that adherens junctions play an important but poorly understood role in regulating the dynamics of insulin secretory vesicles.

## Other proteins that make up the cadherin mediated adherens junctions

**Figure 1:**
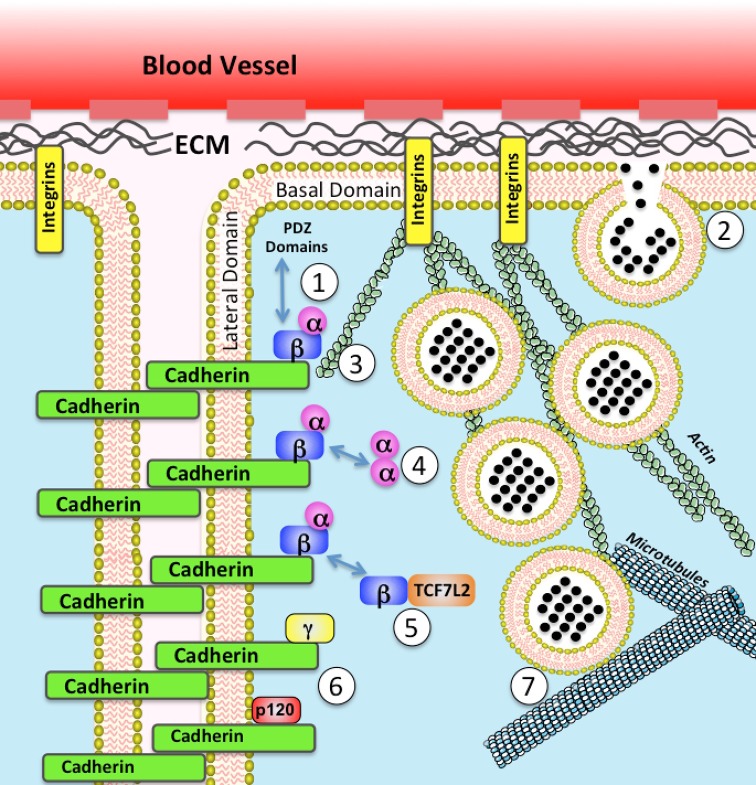
Possible mechanisms by which adherens junction might acutely regulate trafficking of insulin containing vesicles to the basal domain of the plasma membrane 1. Possible interaction with β-catenin with PDZ domains of proteins that are involved in vesicle trafficking; 2. Possible effects on processes affecting vesicle fusion including calcium fluxes and location of components of SNARE complexes; 3. Regulation of actin remodelling controlling vesicle trafficking; 4. Possible regulation of signalling pathways such as hedgehog, YAP/TAZ and NF-κB by α-catenin; 5. Sequestration of β-catenin in nucleus by TCF7L2 altering the balance of proteins at adherens junctions; 6. Possible effects of other catenins that bind to cadherins; 7. Possible regulation of microtubules to control vesicles ability to reach the plasma membrane.

The cytoplasmic domain of cadherins forms a complex that regulates the cytoskeleton by directing the formation of actin filaments beneath the plasma membrane and these play an important role in regulating cell structure [23]. This could provide a mechanism for regulating insulin vesicle trafficking given the importance of actin remodelling in this process as described above. Three major classes of proteins are involved in forming this complex with cadherins (Figure 1). The first of these has two members, these being β-catenin and γ-catenin (also known as plakoglobin). These are closely related proteins whose armadillo repeat regions are both capable of binding to same region in cadherins but each has somewhat different effects on cell function [32]. In both cases this binding to cadherins competes with binding to the transcriptional regulator TCF7L2, an event which results in them being transported to the nucleus to regulate expression of certain genes [32]. In addition, β-catenin has been reported to bind to dynein and so provide a mechanism for adherens junctions to modulate the interaction of microtubules with the plasma membrane [33]. The C-terminal amino acids of β-catenin have also been reported to bind to a range of PDZ domains in proteins known to play key roles in regulating vesicle trafficking although it is not clear what effects these interactions have *in vivo* [34]. A second family of armadillo domain containing proteins that bind to cadherins is the p120 catenin family (p120catenin, δ-catenin, ARVCF and p0071) [35]. These bind to the juxtamembrane regions of N- and E-cadherin which stabilizes them at the plasma membrane [36]. If free in the cytosol, p120-catenin is known to differentially regulate the activity of Rac and RhoA and so contribute to regulation of cytoskeleton and also vesicle trafficking [37]. The third classical component of adherens junctions is α-catenin and there are three isoforms of α-catenin, each expressed in a different range of tissues [23]. These lack armadillo repeats so do not bind directly to cadherins but are localized to the cadherin complexes by binding to the N-terminus of β-catenin and γ-catenin [38,39]. Once detached from β-catenin, the α-catenin can homodimerize and this has a separate set of effects on actin dynamics, in part through regulating actin branching [40].

## The role of catenin’s in the regulation of insulin secretion

**Figure 2:**
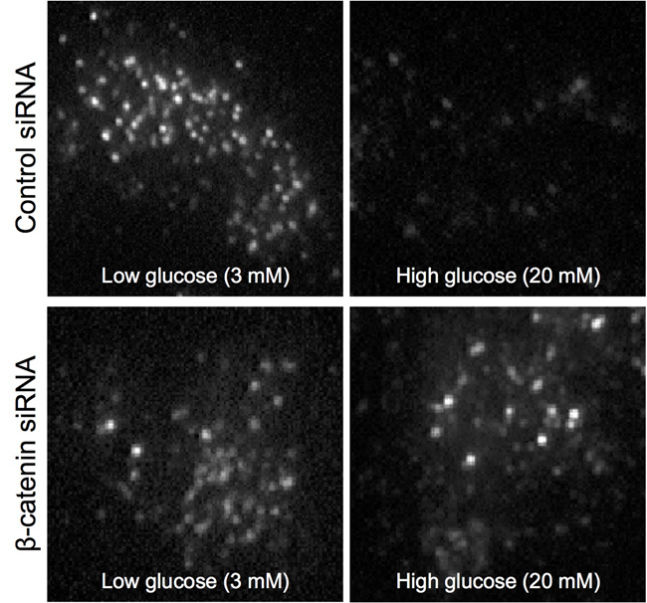
An example using TIRF microscopy in INS1E cells transfected with labelled insulin illustrating how loss of β-catenin leads to redistribution of insulin granules near the plasma membrane and attenuates the release of insulin granules following glucose stimulation (Images produced by Dr Brie Sorrenson and Dr Will Hughes using methods described in [42]).

The findings above highlighted the need to better understand the mechanisms by which adherens junctions regulate trafficking of insulin secretory vesicles. Initial studies have largely focused on β-catenin and show its presence is required for proper GSIS by pancreatic β-cells as depletion of β-catenin using siRNA or reduction in β-catenin level by pyrvinium treatment significantly reduced magnitude of GSIS in rodent pancreatic β-cell lines and mouse islets [41,42]. Furthermore, using TIRF microscopy we found that β-catenin depletion causes the retention of insulin granules at the cell periphery indicating the requirement of β-catenin for insulin granule movement in the final stages of exocytosis [42] (Figure 2). Conversely, incubating cells with the GSK3 inhibitor BIO increases β-catenin levels and as a result of that, GSIS was also increased [42]. Other groups have shown that insulin secretion is increased by treatment of β-cells with Wnts, which will also increase β-catenin [43,44]. The *in vivo* data on the role of β-catenin in insulin secretion are more difficult to interpret given that this is largely based on knockout models as long term loss of β-catenin has multiple effects on cell function and differentiation state. However, mice with β-catenin deleted using the pdx1 promoter to express CRE recombinase have elevated fasting blood glucose and defects in insulin secretion [45]. To overcome the possibility that these defects were the result of defects in pancreas and islet development [46] a model has recently been developed in which β-catenin is largely, but not completely, deleted from β-cells in adult mice using a tamoxifen inducible CRE expressed under the MIP1 promoter [47]. From the limited data presented and the fact the knockout was not complete in this study it is not possible to fully understand the impact of the loss of β-catenin but it highlights an important approach to addressing this issue and detailed analysis of the β-cells in this model would be very informative.

In addition to its role in adherens junctions, β-catenin is also known to play a pivotal role in mediating the effects of the Wnt/Frizzled signalling system and these effects are thought to largely mediated by regulation of gene expression through binding to TCF7L2 to form an active transcription regulatory complex [48]. Therefore, one question was whether this requirement for β-catenin in insulin secretion was via controlling the expression of certain genes. While it seems highly likely that this will play some role in mediating effects of β-catenin in β-cells, our evidence indicates that the acute effects of changes in β-catenin on vesicle trafficking in β-cells are not mediated through gene expression suggesting instead a mechanism involving interactions with adherens junctions at the cell periphery [42]. Consistent with this is the finding that staining of β-catenin in islets shows most of the β-catenin is at the cell periphery in β-cells rather than in the nucleus [47]. We also find that β-catenin depletion in rodent β-cells causes the accumulation of F-actin at the cell periphery and decreases the F-actin/G-actin ratio [42]. This suggests that a cadherin/β-catenin complex could be controlling the cortical cytoskeleton to allow the development of a pool of insulin secretory granules in β-cells that are restrained from fusing with the plasma membrane until a glucose initiated signal is received. Such a scenario is supported by findings in neurons. For example, it has been known for some time that the cadherin-β–catenin complex plays an important role during the recruitment of synaptic vesicles to the synapses [49,50], which is a similar process to insulin vesicle trafficking. When β-catenin is depleted, synaptic vesicles do not accumulate at the synaptic junctions, causing an impaired response to repetitive stimulation [49]. The role of cadherin and β-catenin in regulating actin cytoskeleton in these cells was not investigated but the C-terminal PDZ binding domains of β-catenin is known to be important for β-catenin mediated synaptic vesicle localization [49]. These findings raise the possibility that in addition to any effects mediated through regulating the cytoskeleton that β-catenin may act as a scaffold protein, which recruits PDZ domain containing proteins to the cadherin junction, and may have a role in vesicle localization. In support of this, recent studies indicate that β-catenin interacts with the PDZ domain containing protein scribble, which is an essential regulator of synaptic vesicle turnover [51]. Taken together these results suggest an important role for β-catenin in vesicle trafficking, particularly in establishing the size of the pool of secretory vesicles that are available for rapid release from the β-cell following exposure to secretagogues.

The roles of other components of the adherens junctions in insulin secretion remain to be elucidated. One molecule of particular interest is the β-catenin binding partner α-catenin as there is much evidence indicating both a direct and indirect correlation between α-E catenin remodelling of both microtubules [52] and the actin cytoskeleton [53,54]. In its homodimeric form, α-E catenin binds with actin filaments causing conformational changes, leading to local steric hindrance at Arp2/3 and cofilin binding sites [55]. α-E catenin inhibits barbed end growth, Arp2/3 mediated actin filament branching and severing by cofilin [55]. Importantly, we find that depletion of α-catenin in β-cell models significantly increases GSIS (unpublished data). This would suggest a model where α-catenin is contributing to actin structures that normally suppress insulin secretion. The roles of γ-catenin and the p120 catenins in regulating insulin secretion have not been reported as yet but there is evidence that p120 catenin is involved in regulating vesicle trafficking in adipocytes via its effects on the GTP loading of Rac and Rho [37]. Rac and Rho are also involved in regulating insulin secretion [9].

There remain many gaps in our knowledge of exactly how adherens junctions control insulin secretion. For example, any mechanism explaining how the adherens junction complexes are actually regulating the trafficking of insulin granules has to take in to account the fact the location of the cadherins is in the lateral domains of β-cells while the main site of insulin release appears to be at the basal plasma membrane facing blood vessels [20], although it also remains possible that there is some insulin being secreted in the lateral domains under the control of adherens junctions [21]. One possibility is that the adherens junctions are regulating factors in the lateral domains that are critically required for insulin secretion such as the influx of calcium and that this could then control secretion at the basal surfaces [56]. It could also potentially be explained by long range effects mediated by changes in the cytoskeleton as, for example, there is evidence that actin filaments regulate the function of the SNARE complexes mediating insulin secretory granule fusion with the plasma membrane [57]. Another possibility is that free dimeric α-catenin is acting on the actin at the basal membrane to regulate actin polymerization and that the adherens junctions and β-catenin are acting as a mechanism to regulate levels of free α-catenin in cells. In this vein, it is also possible that the free α-catenin could be activating other signalling pathways that could impact on β-cell function including NF-κB, YAP/TAZ and Hedgehog pathways [58]. In this regard, it is interesting that the hedgehog pathway has been shown to be involved in regulating insulin secretion [59]. Further studies will be required to resolve these issues.

## Physiological implications of adherens junction mediated regulation of insulin release in human disease

The corollary of the finding that adherens junction proteins are required for proper control of insulin secretion is that physiological conditions that alter the levels, functional status, or subcellular localization of the proteins in these complexes are likely to have significant impact on β-cell function. Such changes could potentially represent mechanisms for fine tuning levels of insulin secretion in normal states or could contribute to defective insulin secretion in pathological states. An example of such changes is the finding that exposure of β-cells to high glucose levels rapidly increases E-cadherin in isolated pancreatic β-cells [31]. Further, mice fed a high-fat diet until they became diabetic exhibited increased β-cell mass but a reduced level of GSIS. The islets of these mice also have significantly lower levels of E-cadherin, N-cadherin and α-catenin at cell–cell junctions [60]. This could potentially also explain the increased β-cell mass as it has previously been found that reductions in E-cadherin can trigger β-cell division [28]. It is also interesting to note that the reductions in GSIS that are induced by treatment with the anti-depressant drug fluoxetine (Prozac) [61] are also associated with decreases in E-cadherin levels in β-cells [62]. It has also been observed that mice on a low protein diet have reductions in GSIS that correlate with reductions in β-catenin levels in islets [63]. We see rapid changes in β-catenin levels in β-cell models in response to acute changes in glucose levels [41], which could provide a mechanism that would allow glucose levels to regulate the maximum levels of GSIS over the time frame of minutes and hours. This could potentially be involved in the normal fluctuations in insulin secretory capacity seen over time *in vivo* [2]. It is possible that this regulation is in part mediated by phosphorylation of β-catenin as we see rapid changes in phosphorylation of β-catenin in response to glucose and GLP-1 [41,42]. Together this raises the possibility that changes in levels of adherens junction proteins or their phosphorylation state can contribute to not only normal processes regulating the amount of insulin that can be secreted upon glucose stimulation but also to dysregulation of insulin secretion that occurs with aberrant glucose levels and the switch to β-cell proliferation seen in metabolic syndrome.

There is also evidence that some of the known genetic risk factors for β-cell dysfunction may be acting, at least in part, by regulating adherens junctions in β-cells. One example is evidence that expression of the dominant negative form of HNF-1α that causes MODY3 also causes reduced GSIS in islets and this is associated with reduced expression of E-cadherin [27]. A number of SNPs have been identified in the *TCF7L2* gene and these result in increased levels of the TCF7L2 protein in β-cells [64,65]. TCF7L2 levels are also reported to be increased in β-cells in type-2 diabetics, presumably many of whom do not have the *TCF7L2* SNPs, indicating that dysregulation of *TCF7L2* may be a more general feature of type-2 diabetes [66]. Since TCF7L2 binds to β-catenin and localizes it to the nucleus, high TCF7L2 levels could have an effect on how β-catenin functions by, for example, sequestering β-catenin away from the cadherin complexes at the periphery and/or triggering other processes such as cell division by activating gene expression. In support of such a mechanism we and others find that overexpression of TCF7L2 in β-cell models reduces the level of GSIS [42]. Conversely, it has also been observed that increasing levels of E-cadherin in β-cells resulted in decreased levels of nuclear β-catenin and reduced levels of cellular proliferation [28].

## Summary

It has been realized for some time that cell–cell contacts play a key role in allowing β-cells to develop a mechanism that prevents automatic access of insulin secretory vesicles to the membranes while at the same time facilitating mechanisms that then allow these vesicles to be released to the plasma membrane following glucose stimulation. As discussed in this review, it is now becoming clear that the polarization of β-cells and the formation of cadherin mediated adherens junctions play an important role in the development of a pool of insulin containing vesicles that can be recruited to and fuse with the plasma membrane when glucose levels increase. The exact mechanisms involved remain to be fully defined but there are a number of a number of possibilities worthy of further investigation (Figure 1). This together with the fact that levels of some cadherins and catenins are quite rapidly modulated in response to normal changes in physiological parameters or dysregulated in response to disease relevant states suggests that cadherin mediated adherens junctions represent a previously under appreciated, but vital, component of the overall mechanisms involved in controlling the appropriate regulation of insulin secretion.

## Abbreviations

GSIS: glucose stimulated insulin secretion
TIRF: total internal reflectance fluorescent
